# A study on smart home use intention of elderly consumers based on technology acceptance models

**DOI:** 10.1371/journal.pone.0300574

**Published:** 2024-03-27

**Authors:** Chengmin Zhou, Yawen Qian, Jake Kaner

**Affiliations:** 1 College of Furnishings and Industrial Design, Nanjing Forestry University, Nanjing, Jiangsu, China; 2 Jiangsu Co-Innovation Center of Efficient Processing and Utilization of Forest Resources, Nanjing, China; 3 School of Art and Design, Nottingham Trent University, Nottingham, United Kingdom; Universiti Tenaga Nasional, MALAYSIA

## Abstract

**Purpose:**

Smart home devices have great potential to improve the quality of life and independence of older people, positively impacting their health, safety, and comfort. However, Chinese research in this field is still in its early stages. Therefore, more comprehensive and in-depth studies are needed to comprehend the various aspects influencing the acceptance and use of smart homes by older users.

**Patients and methods:**

This study adopted the Technology Acceptance Model (TAM) and included perceived usefulness, perceived ease of use, usage intention, intergenerational technology support, perceived value, and perceived risk as extension variables to delve deeper into the behavioral intentions of older users in smart home services. The study used a convenience sampling method to randomly distribute 236 questionnaires among older adults over the age of 60 in the school’s community and neighboring urban communities who have experience in smart home use and who can complete human-computer interactions either independently or with the help of others, mainly focusing on the four sections: user characteristics, family situation, experience of use, and usage intention. The study used structural equation modeling (SEM) and factor analysis to analyze the completion of questionnaires. Finally, we conducted a validation analysis of the rationality and scientificity of the model and derived the six dimensions of the model of the influencing factors on the use of smart home products by the elderly and the weight sizes of their corresponding 13 influencing factors.

**Results:**

The results show that perceived usefulness and perceived ease of use have a positive effect on users’ intention to use smart homes. Perceived ease of use has a positive effect on the perceived usefulness of smart homes. In addition, intergenerational technology support, perceived value, and perceived risk impact users’ perceived usefulness and perceived ease of use of the smart home.

**Conclusion:**

This research aims to describe the factors influencing older users’ willingness to use smart homes. The findings are not only significant for the elderly in China but also of broad value to other regions and countries facing similar demographic challenges. The development of smart homes not only involves the elderly but is also closely related to all segments of society. The government should increase policy support and guide more social forces to participate in the development of the smart home industry. Service providers and designers should fully understand the demand situation and user experience of target users to develop easy-to-use smart home solutions. At the same time, smart homes, as intelligent products for the elderly, need to focus not only on the basic needs of the elderly such as material life and home safety, but also on the spiritual needs of elderly users. Children or caregivers should always pay attention to the psychological state of the elderly and actively guide them to use smart homes to help them realize their self-worth. We look forward to more research focusing on this area in the future and further exploring the specific issues and solutions involved.

## Introduction

With the ongoing advancement in medical innovation, the gradual enhancement of the social environment, and the improvement of living standards, the positive trend of human health has been strongly promoted. According to relevant predictions, by the end of 2025, the total number of senior citizens over the age of 60 in China will exceed 300 million, making up more than 20% of the country’s total population [1, 2]. However, this trend is also accompanied by a series of severe elderly-related problems involving many aspects such as pension, medical care, nursing care, culture and entertainment, and social values. In the area of old age, as the pressure on pension payments gradually increases, the demand for nursing homes, care centers, and assisted living services is rising, and individuals need to plan for their retirement to cope with the financial pressure. On the healthcare front, there are challenges in the allocation of healthcare resources, management of chronic diseases, and the burden of healthcare costs. On the nursing side, the demand for long-term care and nursing care, the need for home care support and training, and the shortage of nursing staff have become the focus of attention. On the cultural and recreational front, monotonous activity facilities, limited socialization opportunities, and lack of spiritual care are in dire need of improvement. The issue of the needs of the elderly has attracted the attention of the Government and academia. The government has introduced a series of policy measures to create favorable conditions for the development of the smart elderly industry. The rapid development of the Internet of Things, big data, artificial intelligence, and other technologies has promoted the continuous innovation of smart elderly products and services, which can better meet the diversified needs of the elderly, and China’s smart elderly industry has developed rapidly. However, the development of the smart aging industry also faces some unique challenges. Due to China’s relatively late entry into the aging society, compared with vulnerable groups such as young children, the demand for elderly groups in the service field is not accurately grasped, and there is an imbalance between demand and supply, a single service model, and unclear industrial positioning. The lack of standards and norms for elderly care has led to the development of the industry, which is often characterized by blindness and disorder. In addition, the elderly’s knowledge of smart aging is still weak, coupled with the decline in physical function and self-perception of aging, low acceptance of new things, poor willingness to participate, and other issues, which to a certain extent limits the promotion and application of smart aging products [3–5].

The primary location for providing care for the elderly will be their homes [6, 7]. As they age, older persons face the effects of multiple factors such as reduced mobility, cognitive decline, and one or more chronic diseases, which limit their range of motion and increase the risk of falls and injuries in the home. In addition, they may forget things more easily, their thinking slows down and their ability to deal with complex problems diminishes. Their need for IT support is stronger and rising than that of younger people [8]. Smart home is a system that utilizes advanced means such as sensing technology, computer, and network systems to provide a safe, comfortable, and energy-efficient environment for residences, and it is also a core part of intelligent buildings [9–11]. Smart home systems bring a more convenient and efficient life to the elderly by integrating various advanced technologies and devices, such as voice assistants, intelligent sensors, and automatic control systems. Assisted living sensors and intelligent products can provide smart care services to residents by monitoring and observing their environmental conditions, physical signs, voice, and facial expression data, and even contacting designated caregivers in the event of unusual activity, thus ensuring the safety and independence of residents’ lives [12, 13]. For example, Kidd used a smart home system radio frequency (RF) tagging to guide elderly users to find lost items [14]. Yang developed a hand-tracking system that recognizes gestures and helps the elderly achieve control of household appliances after getting the angle of bending of their fingers through a camera [15]. Xia conducted a field intervention experiment on the quality of sleep of the elderly using a wristband and concluded that bed heating can improve microthermal environments, thereby improving sleep disorders and the well-being of senior citizens in cold environments [16]. Such initiatives fully demonstrate the great potential and practical benefits of smart homes in enhancing the quality of life of older persons and promoting their independent living. Through the use of cutting-edge technologies and devices, smart home systems can automatically track and identify the behaviors of older persons, provide personalized support and assistance, and thus enhance the safety and comfort of older persons. Therefore, it is crucial to stimulate the willingness of older users to use smart homes.

With the advancement of sensor technology, smart homes have achieved more comprehensive information interaction functions, significantly optimizing lifestyles increasing the independence of older people, and bringing positive impacts on the health, safety, and comfort of elderly users. However, in practice, the interaction process of older adults with smart products is diverse and complex, and barriers to the use of information technology may arise [17]. When older people learn new technologies, they usually think about whether the new technology is helpful to their work and can bring convenience to their lives; older people generally have some resistance and negative attitudes toward new innovations, and their adaptability and intention to use them are far less than that of the young and middle-aged population. Therefore, as a smart home with the introduction of new technologies, the first thing that has to be resolved is the technology acceptance issue of elderly users. Since the mid-2000s, academics have conducted in-depth research on the reception and usage intention of emerging technologies for specific groups like the elderly and proposed classic theories such as the technology acceptance model (TAM) and unified theory of technology acceptance and usage (UTAUT) [18, 19]. However, by combining domestic and international studies for smart homes in the senior market, we find that these studies mainly focus on smart care areas such as fall detection, emergency calls, and vital signs monitoring, which are of great significance for the elderly population. However, strictly speaking, these studies do not fall into the category of smart home, as they mainly focus on the health care of the senior citizens rather than how smart home devices affect senior citizens’ quality of life. Few studies have looked into how smart home devices affect aspects of quality of life, safety, and convenience for older adults. Therefore, we need to further investigate the impact of smart home devices on senior citizens to better meet their needs and improve their quality of life [20, 21]. Mani and Pal used the technology acceptance model to indicate the impact of smart home devices on older adults and found that perceived usefulness, perceived interoperability, privacy concerns, service cost, and innovation were highly valued by consumers [22, 23]. Nikou pointed out that technology anxiety triggered by the physiological and psychological features of senior citizens significantly affects the adoption decision of mHealth services [24]. Zhou discovered that perceived ease of use and perceived usefulness of telemedicine services have a substantial effect on the telemedicine behavioral intention of older patients according to an expanded TAM model [25]. Overall, older users’ willingness to use emerging technologies are key factor, while older adults’ characteristics, cost of product services, technical factors, privacy risks, ease of use of the interaction process, and psychological perceived usefulness are potential variables that affect older users’ acceptance and willingness to employ new technologies. All of these studies provide references for the empirical application of the TAM in this paper.

Quantitative research on the acceptance of smart home devices by older adults is not yet mature. Therefore, we need a more comprehensive and in-depth exploration of the elements impacting senior users’ acceptance and use of smart homes in China. To address the above issues, this study introduces the technology acceptance model (TAM) and constructs an extended TAM model by taking the characteristics of older adults, the cost of product services, technological factors and privacy risks, the ease of interaction process, and the psychological perception of usefulness as extended variables, and validates its structure using AMOS software. The intrinsic connection between the potential factors is explored by examining the attitude intention and use of smart home services by elderly users. On this basis, in-depth research on product design methods is more targeted and effective and also enables relevant service providers and designers to fully understand the user characteristics and actual demand situation, to propose practical solutions to improve the utilization rate of potential users. Through an in-depth understanding of the needs and characteristics of elderly users, we will promote the wide application of smart home technology in elderly care, thereby improving the quality of life and safety of the elderly. By realizing the above research objectives, we expect to provide the smart home industry with practical suggestions on how to better meet the needs of elderly users and increase their acceptance, as well as provide valuable reference materials for relevant scholars and researchers.

## Theoretical background and hypotheses

According to the technology acceptance model, this section expands a more comprehensive framework model by taking into account the characteristics of older adults, the cost of the product and service, technological factors and privacy risks, the ease of the interaction process, and the psychologically perceived usefulness as additional considerations, and formulating hypotheses accordingly.

### Underlying model

Much research has found that the technology acceptance model (TAM), which is dependable, may be utilized as a theoretical model to investigate the intention to use different kinds of information and communications technology (ICT) and intelligent systems [26]. The TAM was originally developed by Davis in 1989 and is widely used to characterize consumer acceptance of emerging concepts, ideas, and things, as well as the primary variables influencing acceptance. With the continuous progress of information technology, scholars from several disciplines have successively developed the theory of reasoned action (TRA) [27, 28], the theory of planned behavior (TPB) [29, 30], innovation diffusion theory (IDT) over 20 years [31, 32], uses and gratification (U&G) [33] and other theories have been combined with TAM to continuously improve the theoretical model of TAM. Currently, TAM has become one of the most well-liked and convincing strategies for gaining users’ willingness to embrace information technology, so TAM is chosen as the base model in this paper [34–36].

According to the TAM theory, users’ actual adoption or rejection of an appliance is primarily affected by two factors, namely perceived usefulness (PU) and perceived ease of use (PEOU). Among them, the improvement of perceived ease of use will further improve perceived usefulness, while both of them positively affect users’ willingness to use [37]. In this paper, perceived usefulness reflects the extent to which older users perceive an improvement in their work or life performance when using a smart home system, perceived ease of use reflects the extent to which older users perceive ease of use when using a smart home system, and willingness to use refers to the subjective likelihood that an older user plans to use a smart home [38]. According to the published literature, Choukou has investigated the acceptance and willingness to use smart assisted living technologies among older adults, and the results showed that despite some concerns about the adoption of the technology, older adults found such products useful and were willing to try them out [39]. Alaiad’s study also showed that customer desire to use is significantly positively impacted by perceived usefulness [40]. Furthermore, Bao, in extending the traditional TAM model, found that perceived usefulness, perceived ease of use, and perceived safe home environments had a direct positive correlation on smart home acceptance [41]. King’s study confirmed how perceived usefulness is influenced by perceived ease of use [42].

Therefore, the hypothesis can be formulated:

H1: Perceived usefulness (PU) of smart homes by older users favors the intention to usage intention (UI) of smart homes.
H2: Perceived ease of use (PEOU) of smart homes by older users favors the intention to usage intention (UI) of smart homes.
H3: Perceived ease of use (PEOU) of smart homes by older users favors perceived usefulness (PU) of smart homes.

### Extended variables

In the field of geriatric research, Chen emphasizes that although the TAM is a practical and trustworthy framework, it is important to take into account the physiological and psychological changes experienced by older adults during the aging process when predicting their adoption behaviors toward smart homes [43, 44]. To more accurately explain technology adoption within older persons, Chen proposed the concept of the senior technology acceptance model (STAM) by combining the traditional structure of technology acceptance with age-related health and ability factors [45]. In addition, Venkatesh proposed the unified theory of acceptance and use of technology (UTAUT) based on technology acceptance modeling research, which can effectively access the feelings and experiences of older adults when using products, and thus has a high value of application in the process of evaluating the design of age-adapted products [46]. Finally, Nikou summarized the theoretical assessment criteria in the research literature review on influencing older persons’ adoption and use of new technologies, which are broadly categorized into three types: (i) broad acceptability standards, including technology, privacy risk, and economic factors; (ii) personal characteristics, including individual user characteristics, perceived usefulness, perceived ease of use, and use scenarios; and (iii) sociological criteria, including social inclusion and exclusion [37]. These theoretical assessment criteria are important guides for understanding and promoting the adoption and use of new technologies by older adults.

### Intergenerational technical support (ITS)

In modern societies, where age differences lead to increasing segregation, older adults face many challenges in technology use [47]. Many smart home products require a WiFi connection before setup and use, a step that is already quite difficult for many older adults. In addition, the various buttons and functions of smart homes are also more complicated for many older adults. Because of an absence of technical support and ignorance of the device parameters, the complexity of the operating commands tests the already declining memory, learning ability, and even Mandarin ability of older adults, which may lead them less confident in their capacity to understand how to operate them, thus creating technology anxiety [45, 48–50]. In addition, older adults may encounter challenges in learning to operate the smart home. They may not be able to fully understand the designer’s intent and the feedback provided by the device, making the learning process more complex and difficult, and they may experience the phenomenon of ‘teaching and forgetting’ [51, 52]. Liu’s study confirms that technology manipulation is a key issue in most complex smart home systems [53]. Xue found that older users may be prone to rejection of smart home technologies due to anxiety or nervousness, which in turn may lead to cognitive skepticism about the usefulness and ease of use of the smart home, with a direct negative impact on the intention to use [54]. Due to the richness and complexity of the information processing system, the difficulty of using it increases, which discourages many older people, and thus the issue of technical support for older people from their children has become a growing concern [55]. In the areas of technology use, new media acceptability, and new media expertise, younger generations outperform older individuals. Intergenerational technical help from younger generations to older generations has led to more communication topics and communication opportunities between parents and children. Several studies have shown that the phenomenon of intergenerational technical support in the family is very common in the digital age and significantly influences older individuals’ embrace of technology [56, 57].

Therefore, this study takes children’s intergenerational technical support for older adults’ smart home use as an important independent variable and further explores whether children’s technical guidance and assistance to their parents affects older adults’ experience and acceptance of technology use in the process of using smart homes. This study presents the following hypotheses:

H4(a): Intergenerational technical support (ITS) favors perceived usefulness (PU).
H4(b): Intergenerational technical support (ITS) favors perceived ease of use (PEOU).

### Perceived value (PV)

According to market survey data, we found that the largest obstacle to the uptake of innovative technologies is the upfront cost, followed by privacy issues [58]. Purchasing home products is an important part of the household budget; however, for the majority of consumers, smart home products are of high value, and failure to purchase them can bring serious financial losses to consumers. Therefore, when consumers decide whether to purchase smart home products, the sensitivity to perceived value significantly affects their purchase intention [59]. Perceived value refers to the comprehensive evaluation of the utility of a product or service derived by consumers from the benefits received and the costs paid [60]. Factors such as product quality, price, category, and appearance affect consumers’ perceptions of the value of the product, which directly influences their willingness to buy. Consumers’ willingness to buy is facilitated if they can obtain a satisfactory cost-effectiveness of the product, at which point the cost of the product can be adjusted slightly upwards [61–64]. Generally speaking, the higher income and higher education level of the elderly group will have a more thorough comprehension of the concept of community residential care services, intelligent transformation of home products and their role, so that they are more concerned about the home environment and can provide the necessary financial support. In contrast, low-income or low-education people are at a relative disadvantage in the field of consumption. They tend to have more conservative consumer attitudes and pay less attention to smart products. This view is further supported by other academic studies. According to Jamal’s research, customers’ propensity to shop and perceived value are positively correlated [65]. Chang argues that consumers’ evaluation of product value has a major and direct influence on their purchasing decisions and that the process of making decisions is heavily influenced by perceived value [66].

This study presents the following hypotheses:

H5(a): Perceived value (PV) favors perceived usefulness (PU).
H5(b): Perceived value (PV) favors perceived ease of use (PEOU).

### Perceived risk (PR)

The intelligent home atmosphere is an important component that has to be highlighted since it affects the user’s life directly and has the potential to do major harm in risky circumstances. Hemil integrated the diffusion of innovations theory (IDT) into the TAM model, adding two new crucial components, perceived risk (PR) and trust. The model shows that perceived risk has a substantial impact on perceived usefulness [27]. Perceived risk refers to a customer’s subjective perception of the nature and degree of risk that a particular good may pose when considering its purchase [67]. Since smart homes are still emerging in the minds of most potential buyers, most of them are very cautious when deciding whether to buy a smart home or not. Before making a purchase, they usually deeply analyze and evaluate the usability and ease of use of the smart home, as well as the possible risks associated with the purchase, and make their purchase decision accordingly. Featherman and Sintonen reviewed the ethical issues related to the use of smart home devices by older persons over the past decade and concluded that privacy invasion and security are the main variables influencing older adults’ usage of smart homes [68, 69]. Seniors are usually reluctant to delegate all their uncertainties to machines due to misgivings about their technology [70]. On the one hand, smart homes involve high technologies that are not in the hands of the general public, and when hardware and software systems malfunction, the smart home can quickly go out of control and may cause distress or even harm to users [71]. On the other hand, the smart home system is a product of the era of high data and informatization, relying on the collection of user lifestyle data such as exercise, travel, energy use, etc. to provide appropriate services, the home security system may be an illegal invasion or data leakage and other issues, which makes the user concerned about the use of the information security of the smart home [72, 73]. Numerous studies have shown the significant influence perceived risk has on the adoption of new technology. Holak found that for consumer durables, the perceived risk rises significantly with increasing complexity, which reduces consumers’ purchase intention to a certain extent [71]. Yang’s study confirms that security risk and privacy risks hurt the adoption of smart home services by prospective customers [73]. It can be seen that the presence of perceived risk stimulates consumers’ self-protective behaviors, and the greater the perceived risk, the more detrimental it is to consumers’ positive willingness to use [74, 75].

Therefore, this study presents the following hypotheses:

H6(a): Perceived risk (PR) is detrimental to perceived usefulness (PU).
H6(b): Perceived risk (PR) is detrimental to perceived ease of use (PEOU).

**Fig 1:**
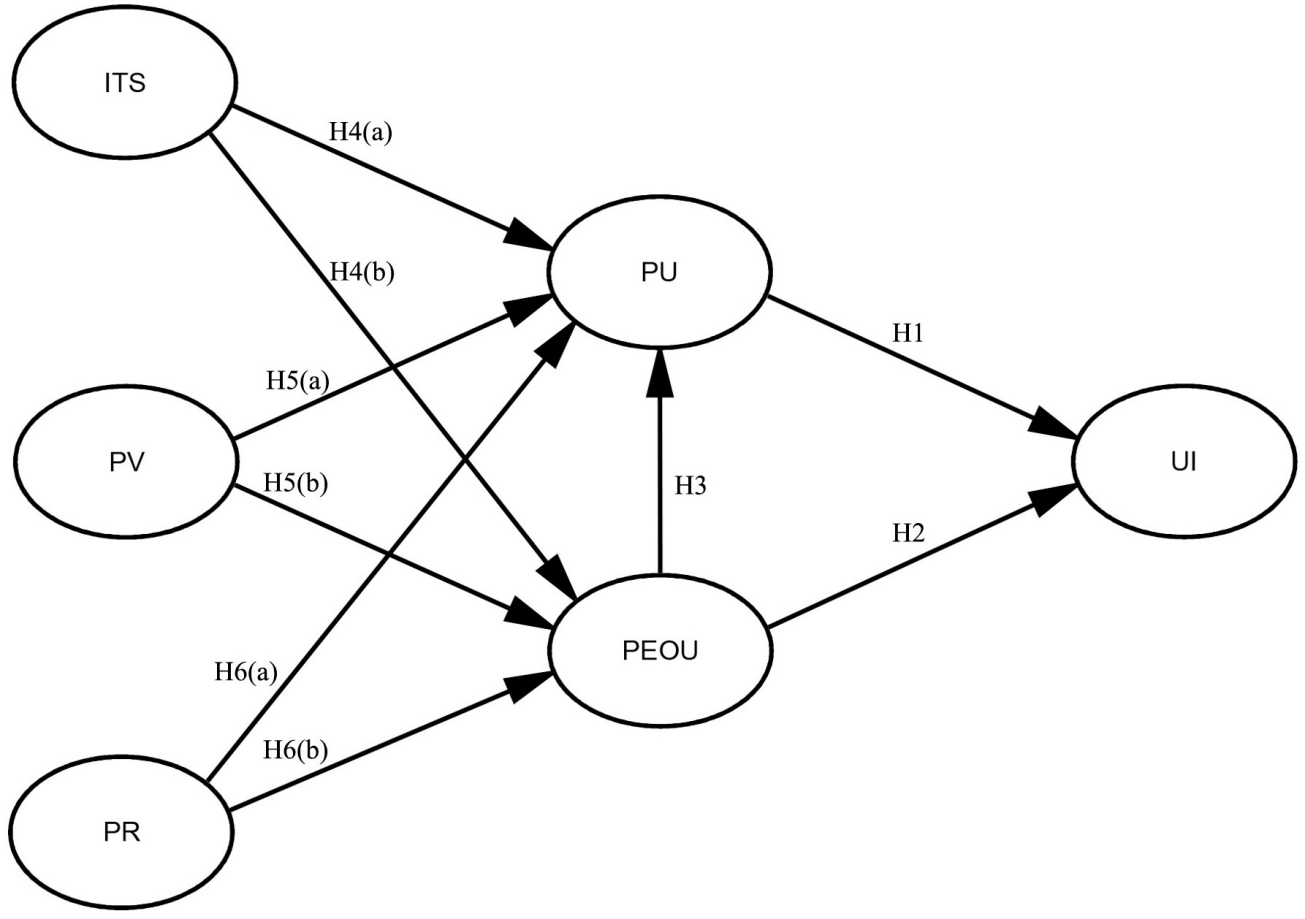
Theoretical model for this study.

Finally, Fig 1 displays the conceptual paradigm suggested in this study.

## Methodology

### Questionnaire design

As a scientific and effective means of modern information acquisition, questionnaire survey is widely used in information collection and analysis. In light of the micro viewpoint, this study utilizes the data obtained from the questionnaire survey to conduct an in-depth discussion on the older population’s desire for smart home devices. In existing studies, we are concerned that the individual variations in the desire for smart homes among the elderly are related to a variety of factors, including their physiological, psychological, cognitive, sociological, and family situations [76].

The questionnaire survey was designed in four parts. The first section mainly collected socio-demographic data about the elderly population, such as age, gender, health status, monthly income, and education level. Some older adults may have difficulty completing basic daily activities due to health conditions or chronic diseases, and smart home devices provide these older adults with the opportunity to continue living at home and preserve their quality of life [77]. The high technology and high investment in smart home devices make the use of smart homes closely related to the income and education level of elderly users and other information. The second part of the study focused on the home situation of the elderly, especially the family structure and living status. It was found that in the case of needing specialized care, the elderly preferred to stay at home rather than enter a healthcare facility, which undoubtedly increased the demand for family members [78]. According to relevant data, China has also been promoting the “9073 model” at the policy level, which means that 90% of the elderly rely on home care, 7% rely on community care, and 3% rely on institutional care. The third section asked respondents about their experience with smart home products. For respondents who have never used the products, we screened them out and terminated the survey. For respondents who had experience in using the products, we continued with the fourth part of the survey, which focused on their views and acceptance of smart home services. All items were assessed utilizing the five-point Likert scale method, which categorizes respondents’ agreement with the content of the question items into five levels: complete disagreement (1), disagreement (2), neutral (3), agreement (4), and complete agreement (5). During the questionnaire, in cases where respondents did not select any option or did not answer a question, the questionnaire was designed to allow for skipping that question and moving on to the next one.

Based on the reference to the relevant mature questionnaires, the fourth part of the questionnaire scale of this study was developed and designed in combination with the previous research, as shown in Table 1. Among them, perceived usefulness, perceived ease of use, and willingness to use mainly refer to the questionnaire of Davis [18]. Among them, perceived usefulness, perceived ease of use, and willingness to use mainly refer to the questionnaire of Davis [18]; perceived usefulness consists of three entries, such as “I think the smart home is a must-have system for life”; perceived ease of use consists of two entries, such as “I think the smart home is easy to learn how to use”; and willingness to use consists of two entries, such as “I will actively use it in the future”; and willingness to use consists of “I will actively use it in the future”. Perceived ease of use consisted of two entries, including “I think smart home is easy to learn how to use”, and willingness to use consisted of two entries, including “I will actively purchase smart home products in the future”. Intergenerational technology support was referenced from questionnaires by Czaja [79], Lie [80], and Slegers [81], and consisted of 2 entries such as “I think my children will guide me in using the smart home”; perceived value was referenced from questionnaires by Hsu [82], Mitzner [83], and consisted of 2 entries such as “I think smart home is easy to learn to use”; willingness to use consisted of 2 entries such as “I will actively purchase smart home products in the future”. “I think the cost of purchasing and using the smart home is acceptable” and 2 other entries; perceived risk refers to the questionnaire of Hemil [31], Alam [84], and Ostlund [85], and consists of, “I think the security performance of the smart home is stable.” and other 2 entries. Both of the 2 entries measuring perceived risk were reverse-scored.

**Table 1:** Measurement scales for variables.

Variable name	Measurement question	Reference source
Perceived usefulness	PU1: I believe that the smart home is a necessary system for life.	[18]
	PU2: I believe that the smart home can better meet my needs.	
	PU3: I believe that the smart home can improve my quality of life.	
Perceived ease of use	PEOU1: I believe that the smart home is easy to learn to operate.	[18]
	PEOU2: I believe it is convenient to live in a smart home.	
Usage intentions	UI1: I will actively purchase smart home products in the future.	[18]
	UI2: I am willing to share smart home products with my friends and family.	
Intergeneration-al technical support	ITS1: I believe my children will guide me in using the smart home.	[79–81]
	ITS2: I believe I could easily operate a smart home with my children’s guidance.	
Perceived value	PV1:I believe the cost of purchasing and using the smart home is acceptable.	[82, 83]
	PV2:I believe that the smart home is worth every penny.	
Perceived risk	PR1: I believe the security of the smart home is stable.	[31, 84, 85]
	PR2: I believe the smart home will not leak my privacy.	

### Participants

According to international regulations, senior citizens are defined as those aged 60 years or older, including young older adults (60 ∼ 74 years old), generally older adults (75 ∼ 90 years old), and long-lived older adults (over 90 years old) [42]. Older persons undergo substantial cognitive changes in comparison to younger adults, including a notable reduction in fundamental cognitive functions like memory and attention. In some cases, memory loss may also lead to a decline in logical reasoning, slower analysis of sensations, and longer reaction times to perform motor behaviors, resulting in a loss of ability to carry out daily tasks. Considering that this study aims to investigate the variables influencing senior users’ inclination to utilize smart home products and that the cognitive abilities involved in human-computer interaction are closely related to attentional management, memory retention, processing speed, executive capacity, and visuospatial ability, this study will focus on elderly people who have experience in using smart homes, are over 60 years old, and can complete human-computer interactions either independently or with the help of others as the main target [86].

### Questionnaire collection

The questionnaire survey for this study was conducted mainly in the school community and nearby cities (i.e., the Yangtze River Delta region), which is easy to implement and less costly. In the survey process, a convenience sampling method was used, i.e., the investigator randomly selected respondents for question-and-answer interactions while encouraging their active participation. Compared with other methods, this method has the advantage of simplicity of operation, while the respondents have a certain degree of randomness. The interviewers used a uniform language of instruction and distributed the questionnaires in the communities where the schools were located and in the surrounding urban communities. For the interviewed elderly with writing ability, we asked them to fill in the questionnaire by themselves; for those who could not fill in the questionnaire by themselves, their primary caregivers or interviewers filled in the questionnaire on their behalf. The questionnaires were collected on-site after completion.

The Ethics Committee of Nanjing Forestry University (Science and Technology Department of Nanjing Forestry University) has evaluated and authorized this study. All interviewees who participated in this study were aware of its background, methodological process, results, and purpose, and had signed a written informed consent or verbally agreed to participate in the study. After completing the research questionnaire, we conducted specific user interviews on the options for the older population. Finally, we analyzed and summarized the research data and proposed feasible smart home product design strategies to increase the willingness of the elderly to use them.

### Data processing

The study began on March 10, 2023, and ended on March 20, 2023, with a total of 236 questionnaires. At the termination of the survey, the collected questionnaires were reviewed and counted. The study was divided into two phases. In the first phase, the questionnaire data were entered into SPSS 26.0 software for preliminary processing. If the percentage of identical answers for an option was 70% or more, or if there was 70% missing data, the sample was considered invalid. After removing the invalid samples, data entry was performed again. In the end, the total number of valid questionnaires was 200, and the effective recovery rate reached 84.75%. For details of the questionnaire contents and responses, please refer to the S1 File. In the second stage, to verify the validity and reliability of the questionnaire, valid data were imported into SPSS 26.0 statistical software for reliability analysis. After this, we used AMOS 26.0 software to build a structural equation model (SEM) and factor analysis to explore the intrinsic connection between various factors affecting the adoption of smart homes by the senior group. We used *α* = 0.05 as the test threshold to guarantee the accuracy and consistency of the analysis findings.

## Results

### Descriptive statistical analysis

Table 2 provides a thorough breakdown of the descriptive statistical analysis results. From the 200 valid samples, it is found that there are more females than males, accounting for 73.5% of the total, while the percentage of males is 26.5%. This is consistent with the characteristics of our national situation, i.e., the average life expectancy of the female population is higher than that of males, and the degree of population aging in females is higher than that of males. Regarding the age distribution, 66.0% of the participants were between 60 ∼ 74 years old, 32.5% were between 75 ∼ 90 years old, and only 1.5% were over 90 years old. Among the respondents, young seniors occupy a major proportion, which suggests that the use of smart homes may be related to age, cognition, mobility, and other aspects. In terms of education level, 53.0% of the participants had an education level of junior high school and above, which indicates that the respondents of this study have a good education level. This good educational background helps elderly users to better understand and operate smart home devices so that they can better take advantage of the convenience that comes with smart homes. The lack of user awareness is a key barrier to China’s present smart home development, which directly leads to a low household penetration rate, and the smart home application environment has not yet been fully diffused. In terms of capacity for self-care, most of the elderly can take care of themselves completely or need only partial help, and only 1.5% of the respondents rely completely on the help of others. As the aging process accelerates, there are more “empty nesters”. Smart home technology can be adapted to the decreasing ability of the elderly to do things, and effectively meet their living needs. In terms of family status, only 7.5% of the respondents live alone, 51.5% live with their spouses, 11% live with their children, and 29.5% live with their spouses and children. After retirement, the elderly have fewer social contacts and a stronger desire for affection, and they need more love and help from their families. Compared to elderly individuals who live alone or have no one to care for them, those who have children to care for them are more likely to be joyful, which in turn affects their attitude towards life. Maintaining good relationships and close ties with family members can reduce the cognitive and learning burden on older adults and encourage them to try new, age-friendly technologies and products. Regarding the experience of using smart home products, over half of the participants said they had familiarized themselves with the basic operating steps, but 21.5% of the respondents still had no knowledge of smart homes. indicated that they were already familiar with the basic operating steps, but 21.5% of respondents were still unaware of the smart home. Despite the growing maturity of AI in the smart home sector, it is still at the stage of promotion and popularization among this older age group.

**Table 2:** Descriptive statistical analysis of demographic variables.

Variable Name	Variable Description	Sample size/PCS	Percentage/%
Gender	Male	53	26.5
	Female	147	73.5
Age	60 ∼ 74 years old	132	66.0
	75 ∼ 90 years old	65	32.5
	>90 years old	3	1.5
Education level	Illiterate	26	13.0
	Primary School	68	34.0
	Junior High School	48	24.0
	High School and above	58	29.0
Monthly income	<1000 yuan	10	5.0
	1000 ∼ 2000 yuan	26	13.0
	2001 ∼ 3000 yuan	80	40.0
	>3001 yuan	84	42.0
Self-care ability	Fully independent	114	57.0
	Need some help	83	41.5
	Fully dependent	3	1.5
Living condition	Individuals living alone	15	7.5
	Living with spouse only	103	51.5
	Living with children only	23	11.5
	Living with spouse and children	59	29.5
Experience	No knowledge	43	21.5
	Understanding	47	23.5
	Familiar	76	38.0
	Mastery	31	15.5
	Proficient	3	1.5

### Reliability and validity analysis

This data involves six dimensions, which are perceived usefulness, perceived ease of use, willingness to use, intergenerational technology support, perceived value, and perceived risk dimensions. The questionnaire data were entered into SPSS 26.0 software, and Cronbach’s *α* coefficients were used to conduct the reliability test, and the results showed that *α* was 0.907. The value of the reliability coefficients of the research data is higher than 0.8, which comprehensively indicates that the data reliability is high. The validity of the questionnaire was analyzed using the kaiser-meyer-olkin (KMO) test and bartlett’s test of sphericity, and the results showed that the KMO was 0.909, and the KMO value was higher than 0.7, which means that the research data can be used to extract information effectively. Meanwhile, bartlett’s test of sphericity (p <0.05) was passed, indicating that the questionnaire has good reliability and validity and that the research data is suitable for factor analysis. As shown in Table 3.

**Table 3:** Scale reliability and validity coefficient tests.

Cronbach alpha coefficient		0.907	
KMO and Barlett test of sphericity	KMO values		0.909
	Barlett test of sphericity	approximate cardinality	1351.653
		df	78
		p	0.000

13 studied items and 6 factors in total underwent confirmatory factor analysis (CFA), which showed good significance as shown in Table 4. The absolute values of standardized loading coefficients (Std. Estimate) were all greater than 0.6 and showed significance, implying a good measurement relationship. The research data reliability coefficient values are all greater than 0.6, which clearly shows that statistical reliability is excellent and suitable for additional study. Meanwhile, the average variance extracted (AVE) value is greater than 0.36 and the component reliability (CR) value is also greater than 0.6, indicating that there is a good control relationship and degree of aggregation between the factors and items. Table 5 implies that it has good discriminant validity.

**Table 4:** Reliability and validity tests of extended TAM questionnaire.

Construct	Item	Coef.	Std. Estimate	alpha	z	p	AVE	CR
PU	PU1	1.000	0.682	0.677			0.413	0.678
	PU2	0.849	0.623		7.091	0.000		
	PU3	0.939	0.621		7.072	0.000		
PEOU	PEOU1	1.000	0.830	0.784			0.646	0.785
	PEOU2	0.953	0.776		12.133	0.000		
UI	UI1	1.000	0.763	0.672	8.830	0.000	0.511	0.676
	UI2	0.827	0.664					
ITS	ITS1	1.000	0.833	0.832	13.573	0.000	0.715	0.834
	ITS2	1.105	0.858					
PV	PV1	1.000	0.845	0.816	13.073	0.000	0.690	0.816
	PV2	1.001	0.816					
PR	PR1	1.000	0.836	0.760	10.408	0.000	0.623	0.767
	PR2	0.999	0.739					

**Table 5:** AVE square root and correlation matrix.

	ITS	PV	PR	PEOU	UI	PU
ITS	0.643					
PV	0.435	0.804				
PR	0.629	0.542	0.715			
PEOU	0.374	0.745	0.477	0.845		
UI	0.469	0.672	0.586	0.653	0.830	
PU	0.383	0.626	0.482	0.605	0.674	0.789

### Model fitness test

**Fig 2:**
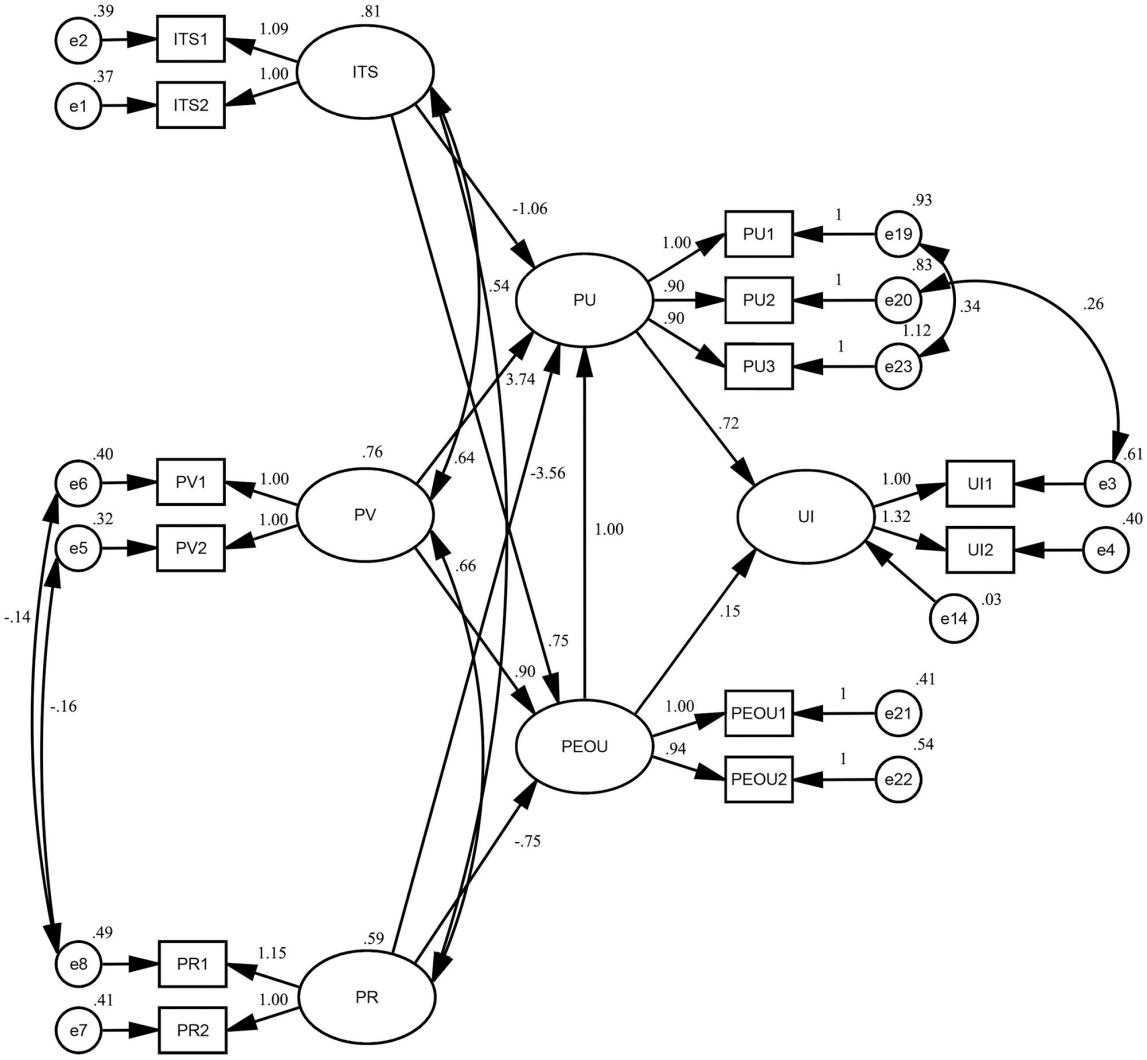
Normalised path coefficient diagram.

The questionnaire data were imported into the AMOS 26.0 software for constructing and testing the measurement model, and path analysis was performed using great likelihood estimation (ML). The indexes and results of the model fit test are shown in Table 6. In the AMOS analysis results, when the chi-square degrees of freedom ratio (CMIN/DF) is 1 ∼ 3, it indicates that the model fit is ideal. The absolute fit index: root mean square error of approximation (RMSEA) value <0.05 indicates acceptable model fit. 3 value-added fit indexes: comparative fit index (CFI), incremental fit index (IFI), and corrective fit index (CFI). Incremental fit index (IFI), and tucker-lewis fit in-Dex (TLI), when their values are in the range of 0 ∼ 1, the closer they are to 1 indicates a higher degree of model fit, and when they are >0.9, they indicate a good model fit. The two parsimony fitness fit indices: parsimony goodness of fit index (PGFI), and parsimony normed fit index (PNFI) are both >0.5, indicating a good model fit. The fitness indicators are shown in Table 6, and in terms of the model fitness test, the CMIN/DF ratio was 1.223, the RMSEA value was 0.033, the CFI value was 0.991, the IFI value was 0.991, the TLI value was 0.987, the PGFI value was 0.546, and the PNFI value was 0.636, and the indicator values were all within the acceptable range. Fig 2 displays the standardized path coefficients.

**Table 6:** Model fitting indexes.

Fitting index	P	CMIN/DF	RMSEA	CFI	IFI	TLI	PGFI	PNFI
Standard value	>0.05	1∼3	<0.05	>0.9	>0.9	>0.9	>0.5	>0.5
Parameter Value	0.130	1.223	0.033	0.991	0.991	0.987	0.546	0.636
Conclusion	Conformity	Conformity	Conformity	Conformity	Conformity	Conformity	Conformity	Conformity

### Hypothesis testing analysis

The model normalized path coefficients reflect the importance of the factors affecting the smart home demand of elderly users, and the path coefficients are normalized and can be regarded as the weighting coefficients of the relevant variables. The SEM path coefficients are shown in Table 7, and the p-value of all the paths is <0.5, which indicates that all the nine hypothetical paths are significant.

**Table 7:** Extended TAM related variables statistic.

Hypothesis	Path	Path coefficient	S.E.	C.R.	P	Result
H1	PU–>UI	0.723	0.177	4.079	***	support
H2	PEOU–>UI	0.151	0.097	1.562	0.118	support
H3	PEOU–>PU	1.000				support
H4(a)	ITS–>PU	-1.056	0.497	-2.126	0.033	unsupported
H4(b)	ITS–>PEOU	0.750	0.158	4.743	***	support
H5(a)	PV–>PU	3.737	2.194	1.703	0.089	support
H5(b)	PV–>PEOU	0.904	0.645	1.402	0.161	support
H6(a)	PR–>PU	-3.563	2.373	-1.501	0.133	support
H6(b)	PR–>PEOU	-0.755	0.695	-1.086	0.277	support

Table 7 displays the impact analysis results on the factors, H1 (PU → UI, *β* = 0.723, p <0.001), H2 (PEOU → UI, *β* = 0.151, p = 0.118), H3 (PEOU → PU, *β* = 1.000), H4(b) (ITS → PEOU, *β* = 0.750, p <0.001), H5(a) (PV → PU, *β* = 3.737, p = 0.089), H5(b) (PV → PEOU, *β* = 0.904, p = 0.161). The six regression coefficients are positive, indicating that there is a positive correlation between all six hypotheses and that an improvement in the status of each variable improves the intention to use the smart home, thus hypothesis 1, hypothesis 2, hypothesis 3, hypothesis H4(b), hypothesis 5(a), hypothesis 5(b) is confirmed. The three regression coefficients of H4(a) (ITS → PU, *β* = -1.056, p = 0.033), H6(a) (PR → PU, *β* = -3.563, p = 0.133), and H6(b) (PR → PEOU, *β* = -0.755, p = 0.277) are negative, which indicates that these three variables have negative effects on the intention to use smart home. There is a negative effect, thus assuming that hypothesis 6(a) and hypothesis 6(b) are confirmed. The results show that all hypotheses H1, H2, and H3 introduced in the base TAM regarding the variables PEOU, PU, and UI are supported, and for the extended variables ITS, PV, and PR, all hypotheses except H4(a) are supported.

## Discussion

By observing the relationship between observable and latent variables, the key factors influencing the use of smart homes by older users can be further identified. The results of the validation of the proposed nine research hypotheses are interpreted as follows.

H1: Perceived usefulness (PU) of smart homes by older users favors the intention to usage intention (UI) of smart homes. According to the coefficient test findings between the factors and measurement terms in the model, we found that the standardized regression coefficient of perceived usefulness on desire to usage is 0.723, and the C.R. value is 4.079, which shows a positive effect. This result indicates that the performance of smart home products affects the willingness to use elderly users to a large extent, verifying the correctness of hypothesis 1. This suggests that elderly customers place a high value on the quality and performance of smart home products, and only high-quality products can win the favor of consumers. As older people’s physical abilities are deteriorating, traditional home products can no longer meet their daily life needs, so targeted assisted living home products are needed. The core goal of a smart home is to provide a safer, convenient, and comfortable living space. However, many smart home products are not designed and developed with the elderly in mind, nor do they fully consider their physiological needs. Despite the rich functionality of smart home products, only a small portion of the functions are practical and convenient for the elderly. Therefore, these products not only fail to realize the real “intelligence”, but even bring a lot of inconvenience to the lives of the seniors. Consequently, the functions of smart home products for seniors ought to be subtracted and based on natural user interaction, including visual interaction, voice interaction, and somatosensory interaction. This can improve the recognition accuracy and enable the elderly to interact with smart homes through natural language.

H2: Perceived ease of use (PEOU) of smart homes by older users favors the intention to usage intention (UI) of smart homes. The standardized regression coefficient of perceived ease of use (PEOU) on willingness to use (UI) is 0.151, which has a positive effect, indicating that the easy operation and powerful functions of smart home products effectively improve the willingness of elderly users to a certain extent, thus verifying Hypothesis 2. Under the “9073” aging pattern, aging in place is the norm, which means that the elderly are one of the groups of people who spend the longest time at home. However, due to the physiological decline of the elderly, their mobility, brain power, visual ability, and ability to cope with the surrounding environment have all been weakened, resulting in a weakening of their ability to control and manage a variety of home equipment. Given the growth of China’s economy and the change in social concepts, young people and the elderly usually live separately, and the elderly have a growing demand for a sense of independence. They prefer undisturbed behavior, so when considering the use of smart home products, elderly customers are very worried about the ease of smart home operation. This requires home products to be simple and practical for the elderly. As smart products usually involve many complex technologies, many functions require tedious parameter settings, which are difficult for the elderly to accomplish. Therefore, we need to take some measures to increase the willingness of elderly users to use smart home products. On the one hand, there is a need to enhance the intelligence of the products to simplify the steps to help the elderly to complete the commands. On the other hand, additional remote management or assistance functions are added to be completed with the help of children or caregivers. At the same time, it is also necessary for companies to give full consideration to the cultural literacy characteristics of the elderly when giving instructions, and provide simple, intuitive, and easy-to-understand instructions to help the elderly operate smart products smoothly. These measures will help increase the willingness of elderly users to use smart home products, thereby improving their quality of life.

H3: Perceived ease of use (PEOU) of smart homes by older users favors perceived usefulness (PU) of smart homes. The path coefficient is 1.000, showing a positive effect, indicating that perceived ease of use significantly and positively affects perceived usefulness, which means that the stronger the ease of use of the smart home, the easier it is to be accepted by users. From the perspective of gerontophysiology, as people age, their recognition and reaction speed when using smart products has decreased, therefore, the elderly tend to take longer than young people to complete the relevant operations using smart products, which may lead to the elderly falling into anxiety, and are prone to negative emotions such as sensitivity and depression. These phenomena suggest that the elderly are in a state of “disablement”, and they may easily reject the smart home due to anxiety or nervousness, which in turn creates cognitive skepticism about the usefulness of the smart home. Therefore, when providing services, IoT smart home products must give full consideration to the ease of use of the elderly, to help elderly users use the products more happily. Seniors should be able to use smart homes as easily as feasible, shortening the time cost and steps of learning for the elderly by simplifying the operation and improving the degree of intelligence of the products, etc., and bringing the ultimate user experience to the elderly through the high intelligence of the products themselves.

H4(a): Intergenerational technical support (ITS) favors perceived usefulness (PU). The path coefficient of intergenerational technology support on perceived usefulness is -1.056, p = 0.033, and the path coefficient is negative, which is inconsistent with the original hypothesis. A notable trend in current society is the gradual rise in the proportion of older adults who live alone, this group, especially seniors who live alone, are often not the primary digital technology users, and their acceptance of this domain is relatively low. Considering the psychological characteristics of the elderly such as high self-esteem and defying old age, letting them do what they can and give play to their potential will make them feel great joy, while some specialized designs dedicated to the elderly group will instead be rejected by them, which in turn will create cognitive doubts about the usefulness of the smart home. Therefore, aging smart homes should fully consider the self-realization needs of senior citizens in the design of new goods and services. To meet this demand, enterprise designers need to fully consider the cognitive, physiological, and behavioral interaction characteristics of the elderly, and effectively apply them to the product interface and operation design to better assist the elderly in completing the operation process and provide a quality experience. The relevant staff ought to be mindful of the intergenerational communication of the elderly in product promotion and operation guidance, work with their children to comprehend the inner demands and subjective feelings of the senior citizens, and provide effective help for the specific difficulties faced by the elderly.

H4(b): Intergenerational technical support (ITS) favors perceived ease of use (PEOU). The path coefficient is 0.750 and positive, so hypothesis H4(b) is confirmed. Older people differ in their family structure and living situation, which leads to differences in their needs for elderly services and subjective feelings about their lives. Older people with children in their care are more likely to have positive emotions compared to older people living alone or without care. Positive intergenerational support and spiritual activities help to relieve the worries of the elderly, which in turn improves their mental health and influences their attitudes toward life. Maintaining good relationships and close ties with family members can reduce the psychological burden of cognitive learning and motivate older people to try new and emerging age-friendly technologies and products. Seniors’ desire to adopt new technologies can be increased by intergenerational technical support from their offspring, which can help them address general technology operating difficulties and reduce their apprehension about technology.

H5(a): Perceived value (PV) favors perceived usefulness (PU). The path coefficient is 3.737, showing a positive effect, and hypothesis H5(a) is confirmed. Perceived value (PV) has the largest path coefficient on the perceived usefulness (PU) of smart home, which indicates that when selecting a product, older customers place a high value on its worth and take into account several aspects, including brand, category, price, and product quality. When consumers perceive the value of smart home products or services to be higher than the cost they pay, such as improving the convenience, safety, and comfort of life, their perceived value of these products or services increases. This perceived value enhances consumers’ positive perceptions of the usefulness of smart homes. This perceivable value can bring pleasure not only before the consumer purchases but also during the process of using the product after purchase. However, certain features of smart products may not apply to older people with different self-care abilities, but adding these features may increase production costs. Therefore, while ensuring quality, companies need to attend to the elderly users’ actual needs level and design products with practical and useful functions that meet the functional needs and sense of value of users from the perspective of the actual needs of customers. This not only reduces unnecessary costs but also meets the price expectations of most people for the product, realizing a win-win situation for both enterprises and consumers.

H5(b): Perceived value (PV) favors perceived ease of use (PEOU). The path coefficient is 0.904 and shows a positive effect, hypothesis H5(b) is confirmed. Perceived value (PV) also has a positive effect on perceived ease of use (PEOU). Perceived ease of use (PEOU) refers to how easy or difficult consumers think it is to use a product or service. When consumers have a high perceived value of smart homes, they are more likely to be willing to invest time and effort to learn and use these products or services. This willingness to invest will make them find smart home products easier to use because they are more willing to overcome obstacles in the process of using them. Overall, perceived value plays a key mediating role in consumer acceptance of smart homes. It not only enhances consumers’ perception of the usefulness of smart homes but also their perception of their ease of use. Therefore, for the smart home industry, enhancing the perceived value of products is the key to improving consumer acceptance and market competitiveness. To achieve this goal, companies can enhance consumers’ perceived value of smart homes by providing high-quality products, optimizing user experience, and strengthening marketing.

H6(a): Perceived risk (PR) is detrimental to perceived usefulness (PU). The path coefficient is -3.563, showing a negative effect, and hypothesis H6(a) is confirmed. It shows that the perceived risk associated with smart home products and their related services for elderly users has a noteworthy adverse effect on perceived usefulness, which in turn inhibits their willingness to utilize smart home products. A possible explanation for this negative impact is that the current aging in China is deepening, and living alone has become the main residence pattern of the elderly. In this state of living alone, residential safety has become the most concerned need of the elderly and their children. Senior consumers may be somewhat concerned about the safety of the use of smart products themselves, the complexity of installation and operation, and the durability of use. Therefore, the security and reliability of smart home service terminals must be ensured to prevent economic loss and personal danger to users. In addition, the data security and privacy issues involved in the aging smart home are also issues that need to be emphasized. Aging smart homes will collect, store, and analyze a vast quantity of various statistics such as the state of the elderly user’s home environment, user status, usage data, etc., to provide more personalized, thoughtful, and detailed, intelligent services to elderly users. However, privacy issues have been controversial when cameras are utilized for gathering information. Consequently, the protection of data security and personal privacy of the aging smart home requires both high attention from the whole society and governmental supervision, as well as continuous deepening of technological research and development to protect it through technical means.

H6(b): Perceived risk (PR) is detrimental to perceived ease of use (PEOU). The path coefficient is -0.755, respectively, showing a negative effect, and hypothesis H6(b) is confirmed. Perceived risk (PR) involves the uncertainty, insecurity, or potential loss that consumers may feel when using a new product or service. For a technological product such as a smart home, consumers may worry about privacy leakage, system security, and operational complexity, and these concerns can affect their perception of the ease of use of the smart home. When consumers perceive risks in using smart homes, they may find these devices difficult to operate or inconvenient to use. This is because consumers may be concerned about safety issues due to their improper operation, or they may need to spend more time and effort to learn and master how to use these devices safely. This perceived difficulty and inconvenience can lead to lower consumer ratings of smart home ease of use. In order to increase consumer acceptance of smart homes, companies need to take measures to reduce perceived risk, such as enhancing product security, providing easy-to-use interfaces, and strengthening user education and support. Through these measures, companies can enhance consumers’ trust and confidence in smart homes, thereby improving their market competitiveness.

## Conclusions

### Conclusions

Against the backdrop of a deepening trend of population aging, the trend of smart homes for aging is becoming more and more obvious. Although smart home appliances have a chance to significantly enhance the lives of the elderly, their popularity is still in its infancy in China, especially among the elderly. Smart home products developed, produced, and designed for the elderly are extremely scarce, and the elderly still face many difficulties in using smart homes. Therefore, we need a deeper comprehension of the various aspects that influence elderly users’ acceptance and use of smart homes.

To explore and validate the aspects influencing older consumers’ use of smart homes and to determine their level of importance, this study employs the technology acceptance model with additional variables. The study used convenience sampling to distribute 236 questionnaires among older adults in the Yangtze River Delta region who are over 60 years old, who have experience in using smart homes, and who can complete human-computer interactions independently or with the help of others. In addition, user interviews were conducted to understand the barriers and specific needs of this population when using smart home services in terms of four aspects: user characteristics, family situation, experience of use, and willingness to use. 200 relevant samples were examined to summarize the study’s findings using structural equation modeling (SEM) and factor analysis.

After the comprehensive data processing and model validation analysis, we derived the six dimensions of the model of influencing factors on the use of smart home products by the elderly and the weight sizes of their corresponding 13 influencing factors. The study’s findings indicate that older users’ propensity to adopt smart homes is greatly affected by their perceived usefulness and perceived ease of use. Meanwhile, there is a significant improvement in the perceived ease of use of smart homes on the perceived usefulness of smart homes by older users. In addition, intergenerational technical support has a significant negative effect on the perceived usefulness of smart homes and a significant positive effect on the perceived ease of use of smart homes. Furthermore, there is a significant positive effect of perceived value on perceived usefulness and perceived ease of use of smart homes. Finally, there is a significant negative impact of perceived risk on perceived usefulness and perceived ease of use of smart homes by older users.

The results of this study provide in-depth insights at the theoretical level for a comprehensive understanding of smart home acceptance willingness among the elderly population. By introducing and expanding the Technology Acceptance Model (TAM), this study not only enriches the existing theoretical framework but also provides new perspectives for research in other related fields by constructing a more comprehensive and detailed model. This contributes to a more in-depth understanding of the acceptance of smart homes by older users and their influencing factors and provides a valuable reference for future research. In addition, this study adopts an empirical analysis method to verify the validity of the extended TAM model by collecting and analyzing data. This not only enhances the credibility and applicability of the theory but also provides empirical support for subsequent theoretical studies.

At the practical level, this study is a significant guide for the progress and promotion of the smart home industry. Our findings are not only significant for the elderly population in the Yangtze River Delta region but also provide valuable lessons for other regions and countries facing similar demographic issues. Moreover, the primary kind of aged care provided by the “9073” design is home care, which means that the elderly spend a relatively long time at home. Given that the physical functions of the elderly are declining, including mobility, cognition, vision, and ability to cope with their surroundings, they are less capable of controlling and managing various home devices. Therefore, we strongly recommend that all relevant stakeholders pay attention to the smart home demands of seniors and take proactive measures. The government should step up policy support to guide more social forces to participate in the legislative support and provide shopping allowances to encourage the elderly to try out smart home devices. Service providers and designers should fully understand the characteristics and actual needs of target users and emphasize the functionality, operability, and user experience of the products to develop easy-to-use smart home solutions. Children or caregivers should always pay attention to the psychological state of the elderly, actively guide the use of smart homes, and teach them patiently in the process of using them, to assist them in realizing their self-worth.

### Limitations

In terms of the limitations of this research, it is crucial to remember that while extensive literature references and summary screening of social phenomena were conducted in determining the evaluation indicators, the assessment of quantification is still highly subjective. Therefore, future research will aim to improve the subjectivity of the assessment and further explore it using more objective methods and experimental data.

In addition, this research has a rather small sample size, only older adults living in the Yangtze River Delta region who are over 60 years old and have experience in using smart homes and can complete human-computer interactions independently or with the help of others are taken as the main target. In the choice of sampling method, convenience sampling was used, which means that interviewers may tend to select respondents who are closer to them or easily accessible, thus having an impact on the precision of sampling. Meanwhile, this is only a typical case and only explores the factors influencing this target group’s willingness to use smart homes for senior citizens within a specific range, limiting the statistical power and generalization of the results. Phenomena such as different economic and cultural development, social inclusion, and different concepts of old age result in diverse strengths strengths, and breadths in the implementation of smart products for the elderly, resulting in different differences among the elderly thus affecting the reasonableness of the experimental data. In future research, the study can be improved through offline and online open-ended questionnaires synchronized collection, by comparing the social environment in the region where the elderly are located, such as the sense of belonging to a social class, social trust, social activities, the government system support and other aspects of the degree of influence of factors to consider.

In addition, the complete home space using the crowd should include infants, youth, middle-aged, elderly, and other people of all ages. Varies age groups have various needs for the use of intelligent home products. When researching intelligent interactive home products, we should consider their generality as a universal product, which needs to meet the common demands of family members, regardless of age, gender, and other distinctions, but also provide precise and personalized services for users of different age groups.

In future research and development, we need to analyze and study these factors more deeply to serve senior users’ demands and promote the popularization and development of smart homes.

### Prospects

At present, the aging smart home market remains in its infancy, with great development potential and broad market prospects, however, it faces many challenges and problems in practical application.

First of all, since the number of senior people who utilize the Internet is comparatively low, the environment in which they live often has imperfect infrastructure problems, resulting in certain difficulties for the elderly to access the Internet at home. Moreover, the cost of Internet access is generally high, and not all elderly people can afford it, so there is a certain threshold for the use of smart homes for seniors. In addition, the supporting services of the smart home are not yet perfect, hardware and software have not yet been fully integrated into their daily life, and the living environment has not been effectively connected.

Secondly, the complexity of the aging smart home standard is one of the major challenges facing the industry. At present, various smart home brand manufacturers have established their ecosystems and follow different standards, resulting in the inability to realize interoperability between products, and the user experience is affected, which is detrimental to the industry’s overall development.

In addition, addressing the characteristics and requirements of older persons, age-appropriate smart homes have emerged to build a friendly home environment. However, the aging-friendly smart home is not cut off from the smart home but is friendly to all groups of people, which can meet the needs of young people and also take care of older persons’ requirements. However, the vast majority of smart home products on the market are not yet able to do this.

In addition, product market awareness is low and household penetration is not high. At present, the market awareness and acceptance of aging-appropriate smart products in China is low, mainly due to the relatively limited literacy of the elderly, insufficient cognition of smart products and experience in using them, as well as the fact that some of the so-called technological aging-appropriate products do not take into account the learning costs of the elderly, which makes the impression of the elderly consumers poorer. There is also an important reason, most of the families living in foreign countries, mainly in single-family houses, for home security, energy control, and other scenes have a stronger demand, while the country has not yet tapped into the immediate need for smart home use of the scene, many families tend to adopt smart homes because of the need to improve their quality of life, which also leads to the user’s awareness of the product is relatively limited.

Therefore, we strongly urge all relevant stakeholders to pay attention to the needs of the elderly population in terms of smart home technology and take proactive measures. Therefore, we strongly urge all relevant stakeholders to take notice of the needs of the senior population in terms of smart home technology and take proactive measures.

From the government’s perspective, policy support should be increased. On the one hand, smart home is an emerging industry, and more social forces should be guided to join in the aging smart home industry through various means, such as increasing the initial investment and financial subsidies; on the other hand, a certain amount of shopping allowances or preferential purchase rights should be provided to guide the elderly population to test buy and try out the smart home equipment.

From the industry’s point of view, it should actively participate in the stipulation of industry standards and try its best to guarantee the interconnection of products of different brands and manufacturers to promote the growth of the whole industry. The elderly smart home in smart home products meet the premise of industry regulations but also need to take into consideration the unique qualities of the elderly. At present, the standard of intelligent products for the elderly is still unclear, which requires the development of elderly smart home evaluation work, and improving the standard of elderly smart home products in continuous debugging. The design of smart home service terminals should be based on our national standards or an industry standard, there is no standardization of product quality is difficult to ensure the quality of its products, and its reliability is bound to be unable to guaranteed.

From the enterprise point of view should dig deep into the actual needs of the elderly, focusing on the flexibility and customizability of the product, the development of easy-to-use smart home solutions for elderly users, to enhance the experience of elderly users, and pay practical attention to the needs of the elderly users of the product to survive in the aging market.

The users involved in aging smart home products include both the elderly and their children, caregivers, etc. The elderly’s learning ability and memory capacity have declined, so the relevant staff should provide clear usage guidelines and specialized training courses to help the elderly and their loved ones and caregivers learn, stimulate the interest of potential users, and increase the utilization rate.

We must also note that the ethical issues brought about by smart home technology, such as privacy protection, data security, and informed consent, are key factors that need to be carefully considered. While promoting the popularization of smart homes, we must ensure that the rights of users are fully protected. We look forward to more research focusing on this area in the future and further exploring the specific issues and solutions involved.

## Supporting information

S1 File Questionnaire survey on the intention of elderly consumers to use smart homes. (XLS)
